# Molecular Characterization of Hotspot Mutations in HER2, BRAF, KRAS, and PIK3CA in Canine Pulmonary Adenocarcinoma from Japan

**DOI:** 10.3390/vetsci13060596

**Published:** 2026-06-18

**Authors:** Asumi Muramatsu, Tomokazu Nagashima, Kazuhiko Ochiai, Amo Ohnuma, Honoka Kawamura, Yukino Machida, Daigo Azakami, Makoto Bonkobara, Toshiyuki Ishiwata, Masaki Michishita

**Affiliations:** 1 Department of Veterinary Pathology, Faculty of Veterinary Science, Nippon Veterinary and Life Science University, Tokyo 180-8602, Japanamoew555_leorigotoffee@outlook.jp (A.O.); ymachida@nvlu.ac.jp (Y.M.); 2 Laboratory of Veterinary Hygiene, Faculty of Veterinary Science, Nippon Veterinary and Life Science University, Tokyo 180-8602, Japan; kochiai@nvlu.ac.jp; 3 Laboratory of Clinical Oncology, Tokyo University of Agriculture and Technology, Tokyo 183-8538, Japan; ft6225@go.tuat.ac.jp; 4 Laboratory of Veterinary Clinical Pathology, Faculty of Veterinary Science, Nippon Veterinary and Life Science University, Tokyo 180-8602, Japan; bonkobara@nvlu.ac.jp; 5 Division of Aging and Carcinogenesis, Tokyo Metropolitan Institute of Gerontology, Tokyo 173-0015, Japan; tishiwat@tmig.or.jp; 6 Center for One Health, One Welfare, Nippon Veterinary and Life Science University, Tokyo 180-8602, Japan

**Keywords:** canine cancer, pulmonary adenocarcinoma, BRAF, HER2, KRAS, precision oncology, targeted therapy

## Abstract

Primary pulmonary adenocarcinoma in dogs is rare, and effective systemic therapies remain limited. To evaluate the molecular basis of potential precision oncology approaches, hotspot mutations in HER2, BRAF, KRAS, and PIK3CA were analyzed in 20 surgically resected canine pulmonary adenocarcinomas and three canine pulmonary adenocarcinoma cell lines. HER2 V659E and BRAF V595E mutations were each detected in 3/20 cases (15%), while KRAS G12V was detected in 1/20 cases (5%). No PIK3CA hotspot mutations were identified. The BRAF V595E mutation was additionally detected in the AZACL2 cell line. Functional analysis demonstrated increased sensitivity of AZACL2 cells to the BRAF inhibitor dabrafenib and MEK inhibitors including trametinib, compared with BRAF wild-type cell lines. These findings support MAPK pathway dependency in BRAF-mutant canine pulmonary adenocarcinoma. The mutation spectrum was broadly consistent with previous reports, suggesting a conserved molecular landscape across geographic regions. Collectively, these data identify BRAF and HER2 alterations as clinically relevant candidates for molecular diagnostics and targeted therapy in canine pulmonary adenocarcinoma.

## Simple Summary

Canine primary pulmonary adenocarcinoma is a rare malignant tumor for which effective systemic treatments are not well established. We analyzed cancer-associated gene mutations in surgically removed canine pulmonary tumors and examined the effects of targeted drugs in cancer cells carrying a BRAF mutation. HER2 and BRAF mutations were detected in a subset of tumors, and cells with a BRAF mutation were particularly sensitive to inhibitors targeting the MAPK signaling pathway. These results suggest that molecular testing for HER2 and BRAF alterations may help identify dogs that could benefit from targeted therapies in the future.

## 1. Introduction

Primary pulmonary tumors are uncommon in dogs and account for less than 1% of all canine neoplasms [1]. The most frequently reported clinical signs include coughing, lethargy, weight loss, and tachypnoea; however, some affected dogs may remain asymptomatic [1,2]. Diagnostic imaging modalities, including thoracic radiography and computed tomography, facilitate the detection of pulmonary nodules, and surgical excision remains the treatment of choice [2,3]. Although chemotherapeutic agents such as vinorelbine, carboplatin, doxorubicin, and mitoxantrone have been used to manage canine pulmonary cancer, standardized and effective treatment protocols have yet to be established [4,5,6,7,8]. Consequently, there is a growing need for novel therapeutic strategies beyond conventional surgery and cytotoxic chemotherapy, including approaches based on the molecular characterization of canine pulmonary tumors.

By contrast, substantial advances have been made in human lung adenocarcinoma, where recurrent driver mutations such as EGFR abnormalities such as exon 19 deletions, L858R, and exon 20 insertions, BRAF V600E, and KRAS G12C have been identified [9]. These discoveries have enabled the implementation of companion diagnostics and the widespread adaptation of precision medicine strategies [10]. For instance, in BRAF V600E-positive lung cancer, combined inhibition of BRAF and MEK achieves superior antitumor efficacy and safety compared with BRAF inhibitor monotherapy [11,12]. These advances underscore the clinical importance of identifying driver mutations to guide targeted therapies.

Consistent with advances in precision oncology in human lung cancer, several genetic alterations have also been reported in canine pulmonary adenocarcinoma, including HER2 protein overexpression [13,14,15], HER2 V659E mutation [13,15], BRAF V600E [13,16,17], KRAS mutations (G12C, G12D, G12V, and G12R) [13,18,19,20], and PIK3CA mutations [13]. Functional studies have shown that HER2 V659E-mutated cells are sensitive to lapatinib and neratinib in vitro, while anti-HER2 monoclonal antibodies exhibit antitumor efficacy in xenograft mouse models with HER2 overexpression [13,21]. Furthermore, clinical trials in dogs with urothelial carcinoma demonstrated that lapatinib induced tumor regression and improved survival, supporting the feasibility of HER2-targeted therapy in veterinary oncology [22]. Nevertheless, the therapeutic potential of inhibitors directed against BRAF, KRAS, and PIK3CA mutations in canine pulmonary cancer remains largely unexplored.

Therefore, further investigation into the genetic alterations driving canine pulmonary adenocarcinoma and their potential as therapeutic targets is warranted. In this study, we analyzed frozen canine pulmonary adenocarcinoma tissues to detect mutations in candidate driver genes, including *BRAF*, *HER2*, *KRAS*, and *PIK3CA*. In addition, we evaluated the antitumor efficacy of inhibitors targeting identified mutations using established canine lung cancer cell lines.

## 2. Materials and Methods

### 2.1. Case Collection

A total of 20 canine pulmonary tumors resected by lobectomy at the Veterinary Medical Center of Nippon Veterinary and Life Science University between 2017 and 2024 were included in this study (Table 1). These cases were referred from multiple veterinary hospitals in the Kanto region of Japan for evaluation and surgical resection of pulmonary tumors. Individual cases had a median age of 12.5 years (range, 6–16 years) and included one intact male, seven neutered males, two intact females, and ten spayed females. Pulmonary adenocarcinoma was detected in the left cranial lobe (n = 5), left caudal lobe (n = 11), right cranial lobe (n = 1), right middle lobe (n = 1), right caudal lobe (n = 1), and right accessory lobe (n = 1). The breeds represented included Miniature Dachshund (n = 3), Chihuahua (n = 3), Toy Poodle (n = 2), Labrador Retriever (n = 2), French Bulldog (n = 1), Pembroke Welsh Corgi (n = 1), Jack Russell Terrier (n = 1), Papillon (n = 1), Shiba Inu (n = 1), Pomeranian (n = 1), Miniature Poodle (n = 1), Miniature Pinscher (n = 1), and Boston Terrier (n = 1). Portions of the resected tumors were collected for genomic DNA extraction from areas macroscopically judged to contain abundant tumor tissue while avoiding regions of hemorrhage and necrosis whenever possible. The sampled tissue minced, snap-frozen in liquid nitrogen, and stored at −80 °C until genomic DNA extraction. The remaining tumor tissues were fixed in 10% neutral-buffered formalin, routinely processed for paraffin embedding, and sectioned for hematoxylin and eosin staining. Histopathological diagnoses were made according to the classification of pulmonary tumors [1]. Corresponding histological sections were reviewed to verify that the sampled regions were enriched for pulmonary adenocarcinoma cells.

### 2.2. Cell Lines and Culture Conditions

Three canine pulmonary adenocarcinoma cell lines, AZACL1, AZACL2, and cPAC-1, were used in this study. AZACL1 and AZACL2, were purchased from the Cosmo Bio Co., Ltd. (Tokyo, Japan). cPAC-1 was provided by the Laboratory of Clinical Oncology, Tokyo University of Agriculture and Technology, and this cell was originally established and cultured as described previously [23]. All the cell lines were maintained in Dulbecco’s modified Eagle medium (FUJIFILM Wako Pure Chemical Corporation, Osaka, Japan), supplemented with 10% fetal bovine serum (Nichirei, Tokyo, Japan) and 1% antibiotics (Nakarai Tesque, Kyoto, Japan) at 37 °C in 5% CO_2_ atmosphere.

### 2.3. Genomic DNA Extraction, PCR, and Sequencing

Genomic DNA was extracted using a ReliaPrep gDNA Tissue Miniprep System (Promega, Madison, WI, USA) according to the manufacturer’s instructions. The concentration and purity of the extracted DNA were determined by an ultraviolet absorption method, resulting in a range of 25–250 ng of DNA per sample. PCR amplification was performed using a Quick Taq HS dye mix (Toyobo, Osaka, Japan). PCR amplification was performed using primers targeting previously reported hotspot regions of canine HER2 V659, BRAF V595, KRAS codons G12, and PIK3CA H1047. Primer sequences are listed in Table 2. Amplification reactions were carried out under the following conditions: initial denaturation at 94 °C for 2 min, followed by 35 cycles of denaturation at 94 °C for 30 s annealing at 55 °C for 15 s, extension at 72 °C for 30 s, and a final extension 72 °C for 1 min. The primer pairs used for amplifying the BRAF, HER2, KRAS, and PIK3CA exons are listed in Table 3. Sequence data were directly determined using an ABI 3100-Avant Genetic Analyzer (Applied Biosystems, Foster City, CA, USA). For sequence analysis, canine HER2 (GenBank accession number: NM_001003217.3), BRAF (GenBank accession number: AY545218.2), PIK3CA (GenBank accession number: LC625864.1), and KRAS (GenBank accession number: XM_038438708.1) was compared using Genetyx software version 15 (Genetyx Corporation, Tokyo, Japan). Detected mutations were independently confirmed by repeat PCR amplification and Sanger sequencing.

### 2.4. Drug Sensitivity Assays

AZACL1 and AZACL2 cell lines were seeded at 5 × 10^3^ and the cPAC-1 cell line at 1 × 10^4^ cells/well on 96-well plates. After 24 h of culture, cells were treated with a fresh culture medium containing six different doses (final concentration: 0.001, 0.01, 0.1, 1, 10, or 100 μM of dabrafenib (Selleck Biotechnology, Kanagawa, Japan), trametinib (Selleck Biotechnology), selumetinib (Selleck Biotechnology), pimasertib (Sellek Biotechnology), or PD318088 (Selleck Biotechnology)) for 48 h. Each living cell was evaluated using a Cell Counting Kit-8 (Dojindo Laboratories, Kumamoto, Japan). IC_50_ values were calculated from dose–response curves.

### 2.5. Statistical Analysis

The results are presented as means ± standard deviation. The Kolmogorov–Smirnov test was used to test for normal distribution, while the *F* test was used to detect equal distribution. Statistical analyses were conducted using Student’s *t*-test and Welch’s *t*-test in R version 4.5.0. A value of *p* < 0.05 was deemed indicative of a statistically significant difference.

## 3. Results

### 3.1. Detection of Hotspot Mutations in Canine Pulmonary Adenocarcinoma

**Figure 1:**
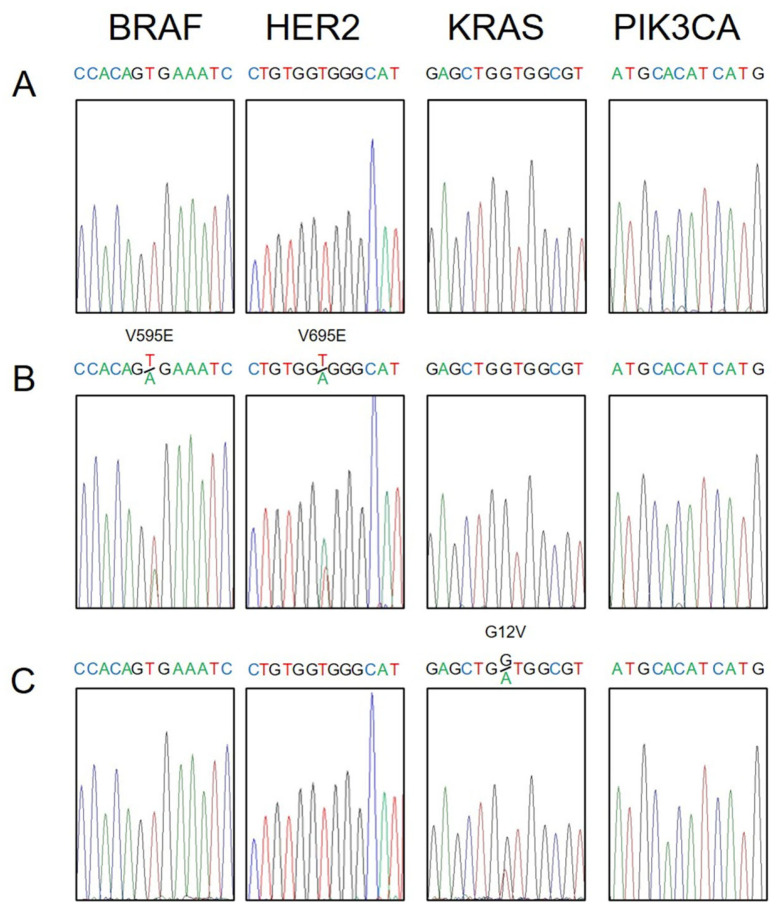
Mutations in *BRAF*, *HER2*, *KRAS*, and *PIK3CA* identified in canine pulmonary carcinoma. (**A**) No hotspot mutations were detected in any of the four analyzed genes in Case 6. (**B**) Hotspot mutations in BRAF V595E and HER2 V659E were identified in Case 19. (**C**) A hotspot mutation in KRAS G12V was detected in Case 14.

Among the 20 canine pulmonary adenocarcinomas analyzed, BRAF V595E mutations were detected in 3 cases (15%), HER2 V659E mutations were detected in 3 cases (15%), and a KRAS G12V mutation was identified in 1 case (5%) (Figure 1, Table 1). No hotspot mutations were detected in PIK3CA. BRAF and HER2 mutations were concurrently identified in the same three tumors.

### 3.2. Mutation Analysis in Canine Pulmonary Adenocarcinoma Cell Lines

**Figure 2:**
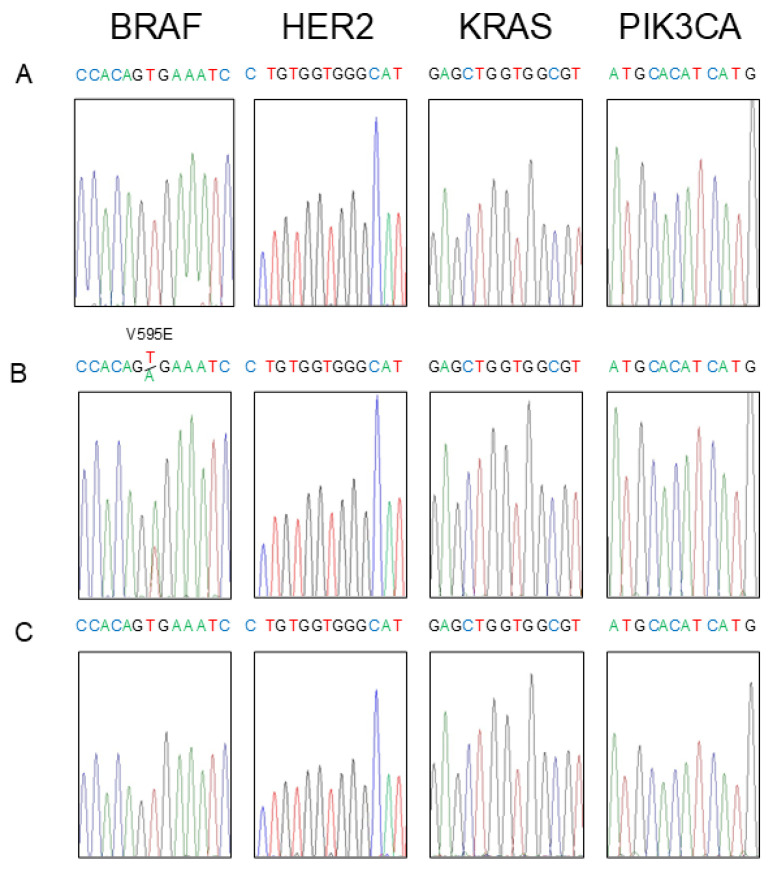
Mutations in *BRAF*, *HER2*, *KRAS*, and *PIK3CA* identified in canine pulmonary carcinoma cell lines. The AZACL2 cell line harbored a BRAF V595E mutation, whereas no mutations were detected in the other three genes analyzed. No mutations were identified in either the AZACL1 or cPAC-1 cell lines. (**A**) AZACL1, (**B**) AZACL2, and (**C**) cPAC-1.

Among the three canine pulmonary adenocarcinoma cell lines, AZACL1, AZACL2, and cPAC-1, examined, the BRAF V595E mutation was detected exclusively in AZACL2 (Figure 2, Table 2). No hotspot mutations in HER2, KRAS, or PIK3CA were detected in AZACL1, AZACL2, or cPAC-1 (Figure 2, Table 3).

### 3.3. In Vitro Sensitivity of Canine Pulmonary Adenocarcinoma Cell Lines to BRAF and MEK Inhibitors

**Figure 3:**
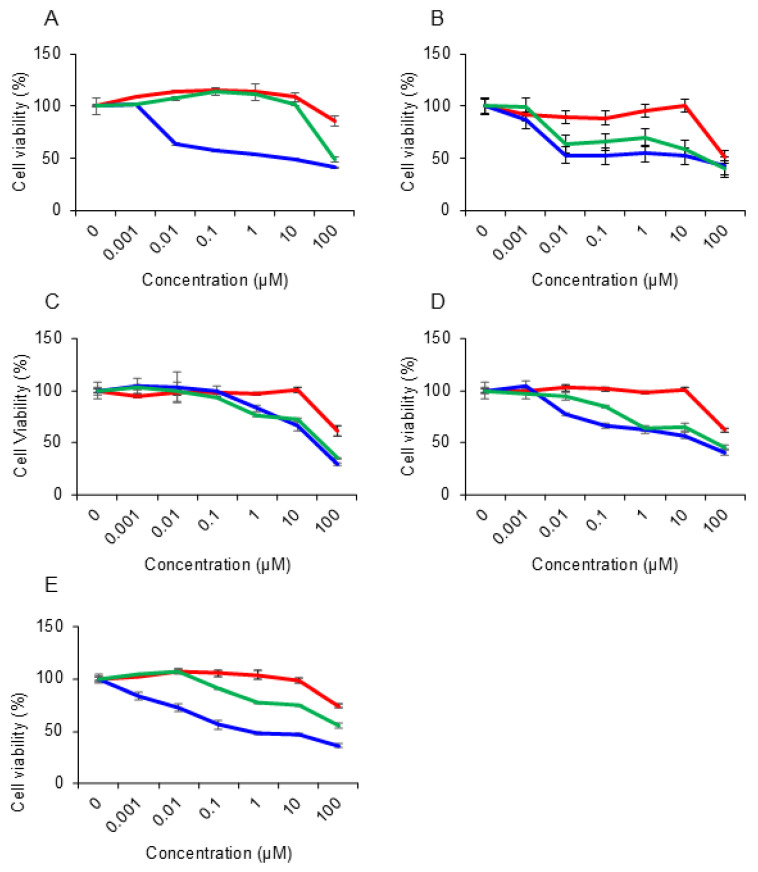
In vitro Drug Sensitivity of Canine Pulmonary Adenocarcinoma Cell lines to BRAF inhibitor dabrafenib and MEK inhibitors. In vitro sensitivity assay of BRAF inhibitor dabrafenib (**A**), MEK inhibitors including trametinib (**B**), selemetinib (**C**), pimasertib (**D**) and PD318088 (**E**), in canine pulmonary carcinoma cell lines, AZACL1 (red line), AZACL2 (blue line), and cPAC-1 (green line). The results are representative of at least three independent experiments. Data are calculated as the mean ± SD.

Drug sensitivity assays demonstrated that AZACL2 cells harboring the BRAF V595E mutation were markedly more sensitive to BRAF inhibitor dabrafenib and MEK inhibitors such as trametinib, selumetinib, pimasertib, and PD318088 than BRAF wild-type AZACL1 and cPAC-1 cells. The IC_50_ values for dabrafenib, trametinib, selumetinib, and pimasertib in AZACL2 cells were 6.59 μM, 14.69 μM, 27.24 μM, and 15.18 μM, respectively, whereas those in cPAC-1 cells were 87.28 μM, 30.66 μM, 40.77 μM, and 58.52 μM, respectively (Figure 3). The IC_50_ value for PD318088 was 79.18 μM in AZACL2 cells but could not be determined in cPAC-1 cells. In AZACL1 cells, IC_50_ values could not be determined for any of the five inhibitors (Figure 3). Overall, the AZACL2 cell line harboring the BRAF V595E mutation exhibited greater sensitivity to BRAF and MEK inhibitors than AZACL1 and cPAC-1 cells, which lack this mutation. These findings indicate enhanced dependency of BRAF-mutant cells on MAPK pathway signaling.

## 4. Discussion

In canine pulmonary cancer, the BRAF V595E mutation has been reported at a relatively low frequency of approximately 5.5% (1/18 cases) [16]. In the present study, however, this mutation was identified in three pulmonary adenocarcinomas and one established cell line, indicating a higher prevalence in our cohort. The BRAF V595E-mutated AZACL2 cell line exhibited greater to dabrafenib and, to a lesser extent, trametinib than BRAF wild-type cell lines, supporting a role for MAPK pathway activation in the proliferation of BRAF-mutant canine pulmonary carcinoma cells. Consistent with these findings, dabrafenib has been shown to modulate oncogenic signaling in canine cancer cells harboring BRAF V595E. Although partial resistance was observed in the canine urothelial carcinoma Love cell line, dabrafenib effectively suppressed ERK phosphorylation, indicating that BRAF inhibition can attenuate downstream-promoting signaling in BRAF-mutant cancers in dogs [24]. On the other hand, trametinib has demonstrated potent antitumor activity in veterinary oncology models. Trametinib significantly inhibited cell proliferation in canine prostate carcinoma cell lines in vitro and suppressed tumor growth in murine xenograft models in vivo [25]. Furthermore, trametinib induced tumor regression in dogs with spontaneous squamous cell carcinoma, highlighting its potential clinical utility as a molecularly targeted therapeutic agent in canine cancers [26]. In human oncology, combined inhibition of BRAF and MEK has been shown to improve therapeutic efficacy and reduce paradoxical MAPK pathway reactivation compared with BRAF inhibitor monotherapy in patients with metastatic melanoma [27]. In lung cancer, dual BRAF and MEK inhibition is an established standard therapeutic strategy for patients with BRAF V600E–mutated non-small cell lung cancer, as reflected in international clinical guidelines [11,12]. These findings support further investigation of MAPK pathway inhibition as a potential therapeutic strategy for BRAF-mutant canine pulmonary carcinoma.

The HER2 V659E mutation has been reported in 26–38% of canine pulmonary adenocarcinomas [13,28]. Although the prevalence observed in the present study was comparatively lower (15%), these findings nonetheless highlight the potential importance of HER2 signaling in canine pulmonary tumorigenesis. This mutation, located within the transmembrane domain of HER2, promotes constitutive receptor dimerization and activation, leading to persistent stimulation of downstream MAPK and PI3K/AKT signaling cascades [13]. Previous studies have demonstrated that cancer cells harboring HER2 mutations in dogs are sensitive to the tyrosine kinase inhibitors lapatinib and neratinib, and that anti-HER2 monoclonal antibodies exert antitumor effects in xenograft models [21]. Moreover, a clinical trial in dogs with HER2-positive urothelial carcinoma showed that lapatinib induced tumor regression and prolonged survival [22]. Collectively, these findings suggest that HER2 V659E represents a promising therapeutic target in canine precision oncology, analogous to HER2-driven human lung and breast cancers [12,29].

Interestingly, all three tumors harboring the BRAF V595E mutation also carried the HER2 V659E mutation. Although the small number of cases precludes definitive conclusions, this finding raises the possibility that concurrent activation of HER2- and BRAF-mediated signaling pathways may contribute to tumor development in a subset of canine pulmonary adenocarcinomas. Because both alterations can promote MAPK pathway activation, tumors harboring concurrent mutations may exhibit distinct biological behavior or therapeutic vulnerabilities compared with tumors carrying either alteration alone. In human oncology, co-occurring oncogenic alterations can influence sensitivity and resistance to targeted therapies. Therefore, further studies will be required to determine whether concurrent HER2 and BRAF mutations have implications for prognosis or for the selection of HER2- and MAPK-targeted therapeutic strategies in dogs.

In the present study, a KRAS G12V mutation was detected in only one case (5%). In a larger cohort analysis comprising 88 canine pulmonary adenocarcinomas, KRAS mutations were identified at a frequency of 4.5%, including G12V, G12D, and Q61K variants [13]. In contrast, an earlier genetic analysis of 21 canine non-small cell lung carcinomas including adenocarcinoma, adenosquamous carcinoma, and large cell carcinoma reported a higher prevalence of KRAS G12 mutations (20%, 4/20 cases), with G12D, G12C, G12V, and G12G variants identified [18]. Similarly, Mochizuki and Breen [16] detected KRAS mutations in 11% (2/18 cases) of canine pulmonary carcinoma with both cases harboring the G12D variant. On the other hand, KRAS mutations occur in approximately 14.5% (143/986 cases) of human lung adenocarcinomas, representing one of the most common oncogenic drivers in this disease [9]. The relatively low frequency of KRAS mutations in canine pulmonary cancers compared with human lung cancers may reflect species-specific differences in tumorigenic mechanisms or disparities in environmental mutagen exposure. Nevertheless, KRAS mutations, particularly the G12C variant, have recently become actionable targets in human oncology through the development of covalent small-molecule inhibitors such as sotorasib and adagrasib [30,31]. Future studies involving larger canine cohorts will be required to more precisely define the spectrum and prevalence of KRAS mutations in canine pulmonary cancer and to evaluate the potential applicability of KRAS-targeted therapies within the context of veterinary precision medicine.

Several limitations of this study should be acknowledged. First, the sample size was relatively small, which may limit the generalizability of the findings. Therefore, the results should be interpreted with caution until validated in larger independent cohorts. Second, although the cases were referred from multiple veterinary hospitals in the Kanto region of Japan, all samples were collected and analyzed at a single institution, and the retrospective nature of the study may have introduced selection bias. In addition, all tumors included in this study were obtained following surgical resection. Therefore, the study population may be enriched for dogs with operable pulmonary adenocarcinomas and may underrepresent advanced-stage, metastatic, or non-resectable tumors. Consequently, the mutation frequencies reported herein may not fully reflect the molecular landscape of the entire canine pulmonary adenocarcinoma population. Third, molecular analyses were restricted to previously reported hotspot mutations in HER2, BRAF, KRAS, and PIK3CA. Therefore, additional genomic alterations potentially involved in the pathogenesis of canine pulmonary adenocarcinoma may not have been detected. Third, molecular analyses were restricted to previously reported hotspot mutations in HER2, BRAF, KRAS, and PIK3CA. Therefore, additional genomic alterations potentially involved in the pathogenesis of canine pulmonary adenocarcinoma may not have been detected. In particular, alterations affecting the PI3K/AKT/mTOR signaling pathway were not comprehensively evaluated beyond PIK3CA hotspot mutations. The PI3K/AKT/mTOR pathway plays a central role in regulating cell proliferation, survival, metabolism, and tumor progression in canine cancers [32]. Recent studies have demonstrated activation of this pathway in canine pulmonary carcinoma and have shown antitumor effects of mTOR-targeted therapies in preclinical models [23]. These findings suggest that aberrant PI3K/AKT/mTOR signaling may contribute to canine pulmonary tumorigenesis even in the absence of detectable PIK3CA hotspot mutations. Comprehensive genomic and molecular profiling will therefore be necessary to further characterize the molecular landscape and therapeutic vulnerabilities of canine pulmonary adenocarcinoma. Another limitation is that clinical outcome data, including overall survival, disease progression, and response to therapy, were not available for systematic analysis. Therefore, associations between specific genetic alterations and clinical outcomes could not be assessed in the present study. Future studies incorporating longitudinal clinical follow-up will be necessary to determine the prognostic and therapeutic significance of these mutations. The biological significance of concurrent HER2 V659E and BRAF V595E mutations could not be fully assessed. Because hotspot sequencing was employed, it was not possible to determine whether these mutations were present within the same tumor cell population or in distinct subclonal populations. In addition, functional analyses were performed using a limited number of canine pulmonary carcinoma cell lines, including only a single BRAF-mutant cell line, and no cell lines harboring concurrent HER2 and BRAF mutations were available for study. Therefore, the drug sensitivity observed in AZACL2 may not fully reflect the therapeutic responses of tumors harboring concurrent HER2 and BRAF mutations. Finally, the drug sensitivity experiments were conducted exclusively under in vitro conditions, and no in vivo validation was performed. Therefore, the clinical relevance of the observed responses, particularly for MAPK pathway inhibitors, remains uncertain. In addition, the IC_50_ values observed for several MEK inhibitors were relatively high, and it remains unclear whether such concentrations can be achieved in clinical settings. Therefore, these findings should be interpreted primarily as evidence of in vitro biological activity. Further pharmacokinetic, pharmacodynamic, and in vivo studies will be required to determine whether therapeutically effective drug concentrations can be achieved in canine patients and to establish the clinical utility of molecularly targeted therapies for canine pulmonary adenocarcinoma.

Collectively, the present findings highlight the molecular heterogeneity of canine pulmonary adenocarcinoma and indicate that recurrent HER2 and BRAF alterations occur in a subset of tumors. While the biological and clinical significance of these alterations requires further investigation, the present study expands the current understanding of the molecular landscape of canine pulmonary adenocarcinoma and provides a basis for future studies exploring molecularly guided diagnostic and therapeutic strategies in this disease.

## 5. Conclusions

Canine pulmonary adenocarcinoma exhibits recurrent actionable alterations, particularly BRAF V595E and HER2 V659E. BRAF-mutant cells show enhanced sensitivity to MAPK pathway inhibition, supporting molecularly guided therapeutic strategies in veterinary oncology.

## Figures and Tables

**Table 1:** Case information used in this study.

Case No.	Breed	Location	Age (Years)	Sex	Genetic Mutations			
					*BRAF*	*HER2*	*KRAS*	*PIK3CA*
1	Miniature Dachshund	Left caudal	16	MN	ND	ND	ND	ND
2	Pembroke Welsh Corgi	Accessory	13	MN	ND	ND	ND	ND
3	Chihuahua	Left caudal	10	MN	V595E	V659E	ND	ND
4	French Bulldog	Left cranial	10	MN	V595E	V659E	ND	ND
5	Papillion	Left caudal	16	F	ND	ND	ND	ND
6	Jack Russell Terrier	Left caudal	10	FN	ND	ND	ND	ND
7	Miniature Dachshund	Left caudal	10	M	ND	ND	ND	ND
8	Chihuahua	Right middle	12	FN	ND	ND	ND	ND
9	Labrador Retriever	Left cranial	10	FN	ND	ND	ND	ND
10	Toy Poodle	Left caudal	13	MN	ND	ND	ND	ND
11	Labrador Retriever	Right cranial	13	FN	ND	ND	ND	ND
12	Boston Terrier	Left caudal	15	MN	ND	ND	ND	ND
13	Miniature Pinscher	Left caudal	13	FN	ND	ND	ND	ND
14	Shiba-Inu	Left caudal	12	FN	ND	ND	G12V	ND
15	Miniature Dachshund	Left caudal	13	FN	ND	ND	ND	ND
16	Chihuahua	Left caudal	6	FN	ND	ND	ND	ND
17	Pomeranian	Right caudal	10	MN	ND	ND	ND	ND
18	Miniature Poodle	Left caudal	13	FN	ND	ND	ND	ND
19	Toy Poodle	Left caudal	13	FN	V595E	V659E	ND	ND
20	Border Collie	Left caudal	9	F	ND	ND	ND	ND

Abbreviations: F, female; FN, neutered female; M, male; MN, neutered male; ND, not detected.

**Table 2:** Primer sequences for detection of canine gene mutation.

Gene	Forward	Reverse
*PIK3CA*	5′-CTCAATGATGCTTGGCTCTGG-3′	5′-CTAATGCTGTTCATGGATTGTG-3′
*KRAS*	5′-AAGGTGTTGATAGAGTGGGTT-3′	5′-GAAGAGCTCTCCAGATCTCT-3′
*BRAF*	5′-CACATATGCCAAATAGAACC-3′	5′-GTAGCACCTCAGGGTCCAAA-3′
*HER2*	5′-GGTTGGGCATCGCTCCACTAGG-3′	5′-GTGATCCCTGGATCTTGAGATC-3′

**Table 3:** Gene mutations in canine pulmonary adenocarcinoma cell lines.

Cell Lines	*BRAF*	*HER2*	*KRAS*	*PIK3CA*
AZACL1	ND	ND	ND	ND
AZACL2	V595E	ND	ND	ND
cPAC-1	ND	ND	ND	ND

ND, not detected.

## Data Availability

The original contributions presented in this study are included in the article. Further inquiries can be directed to the corresponding author.

## Abbreviations

The following abbreviations are used in this manuscript:

BRAF	B-Raf proto-oncogene, serine/threonine kinase
HER2	Human epidermal growth factor receptor 2
KRAS	Kirsten rat sarcoma virus
PIK3CA	Phosphatidylinositol-4,5-bisphosphate 3-kinase catalytic subunit alpha
MAPK	Mitogen-Activated protein kinase
